# Overview of oxidative stress and inflammation in diabetes

**DOI:** 10.1111/1753-0407.70014

**Published:** 2024-10-22

**Authors:** Roni Weinberg Sibony, Omri Segev, Saar Dor, Itamar Raz

**Affiliations:** ^1^ Faculty of Medicine Ben‐Gurion University of the Negev Beer Sheva Israel; ^2^ Faculty of Medicine Tel Aviv University Tel Aviv Israel; ^3^ Faculty of Medicine Hebrew University of Jerusalem Jerusalem Israel; ^4^ Diabetes Unit, Department of Endocrinology and Metabolism Hadassah Medical Center Jerusalem Israel

**Keywords:** inflammation, insulin resistance, OS, type 2 diabetes mellitus

## Abstract

The global prevalence of diabetes has increased significantly, leading to various complications and a negative impact on quality of life. Hyperglycemia hyperglycemic‐induced oxidative stress (OS) and inflammation are closely associated with the development and progression of type 2 diabetes mellitus (T2D) and its complications. This review explores the effect of T2D on target organ damage and potential treatments to minimize this damage. The paper examines the pathophysiology of T2D, focusing on low‐grade chronic inflammation and OS and on their impact on insulin resistance. The review discusses the role of inflammation and OS in the development of microvascular and macrovascular complications. The findings highlight the mechanisms by which inflammatory cytokines, stress kinases, and reactive oxygen species (ROS) interfere with insulin signaling pathways, leading to impaired glucose metabolism and organ dysfunction. Lifestyle interventions, including a balanced diet and exercise, can help reduce chronic inflammation and OS, thereby preventing and controlling T2D and its associated complications. Additionally, various antioxidants and anti‐inflammatory agents show potential in reducing OS and inflammation. Some anti‐diabetic drugs, like pioglitazone, metformin, and glucagon‐like peptide‐1 (GLP‐1) agonists, may also have anti‐inflammatory effects. Further research, including randomized controlled trials, is needed to evaluate the efficacy of these interventions.

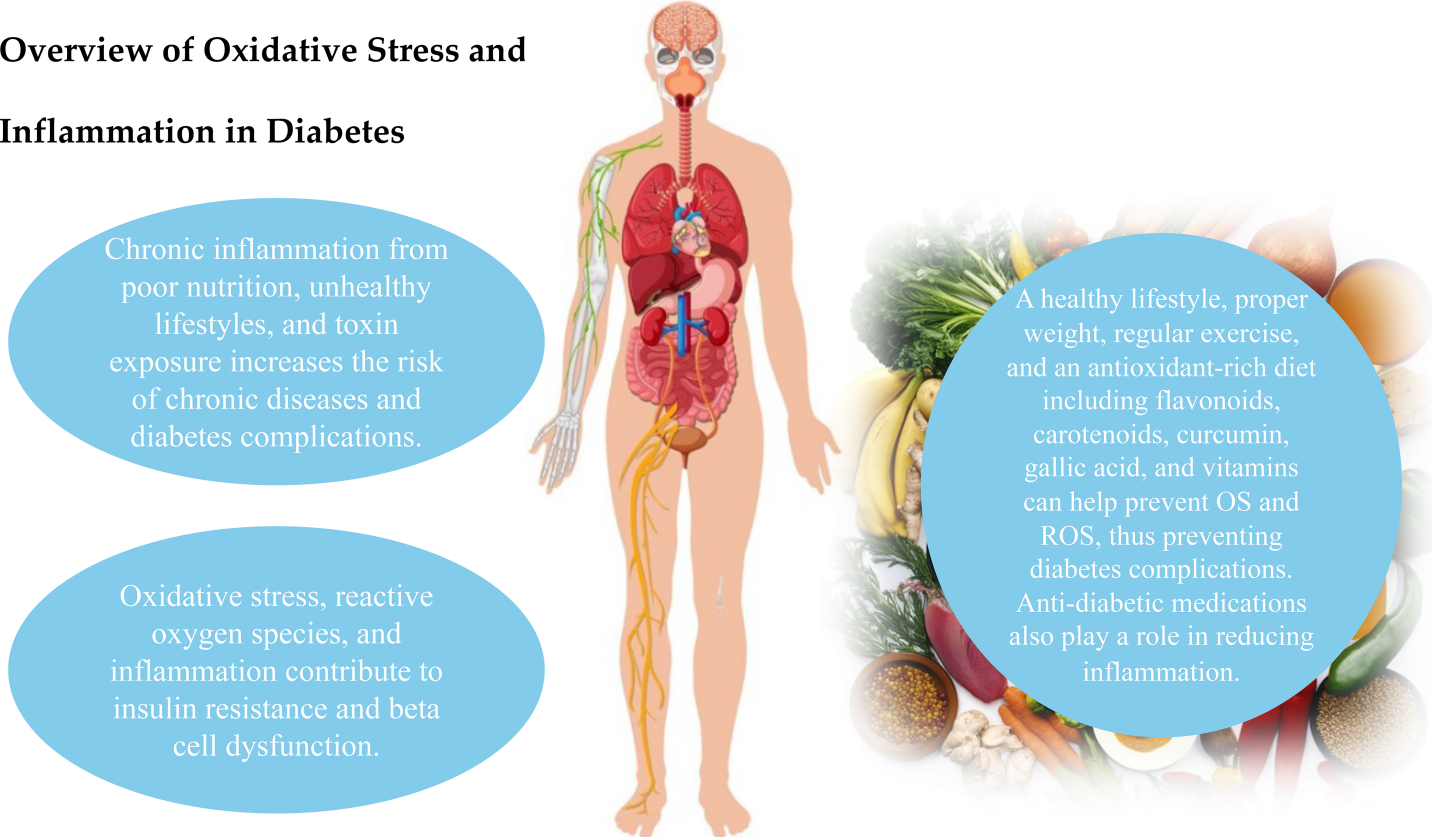

## Graphical abstract

Highlights

- Chronic inflammation from poor nutrition, unhealthy lifestyles, and toxin exposure increases the risk of chronic diseases and diabetes complications.
- OS, ROS, and inflammation contribute to insulin resistance and β‐cell dysfunction.
- A healthy lifestyle, proper weight, regular exercise, and an antioxidant‐rich diet including flavonoids, carotenoids, curcumin, gallic acid, and vitamins can help prevent OS and ROS, thus preventing diabetes complications.
- Anti‐diabetic medications also play a role in reducing inflammation.

## 1 INTRODUCTION

In 1980, the worldwide prevalence of diabetes was approximately 5%. Since then, the global prevalence of diabetes has increased significantly. According to the International Diabetes Federation, it is now 10.5%. It is predicted that by 2045, the total number of individuals with diabetes will be 783 million, representing a prevalence rate of 12.2%.^1^

Diabetes is associated with a range of complications, including microvascular diseases such as retinopathy, nephropathy, and neuropathy, as well as macrovascular diseases, including stroke, myocardial infarction, and peripheral artery disease. Other associated conditions include heart failure and non‐alcoholic fatty liver disease (NAFLD), and there is evidence to suggest that diabetes may contribute to the progression of dementia and Alzheimer's disease. These complications can have a significant impact on the quality of life and can lead to premature mortality. There is a strong correlation between hyperglycemia, hyperglycemic‐induced OS, inflammation, and the development and progression of T2D, as well as its associated complications.^2^

Oxidative stress and inflammation are vital physiological processes with protective roles. Oxidative stress, through reactive oxygen species (ROS), helps eliminate pathogens and signal tissue repair. Inflammation responds to injury or infection by isolating and removing harmful stimuli and initiating healing. However, excessive activation of these processes can lead to diseases. Proper regulation of these processes is essential for preventing and treating related illnesses.

The process of oxidative stress (OS) promotes the generation of inflammatory mediators, which in turn enhance the production of reactive oxygen species (ROS). Excessive production of ROS can be detrimental to cells, causing damaging key cellular constituents such as proteins, lipids, and DNA.^3^ Hyperglycemia is believed to trigger the formation of free radicals and to disrupt the endogenous antioxidant defense systems through various mechanisms.^4^ ROS encompass a diverse array of oxidant molecules that serve multifaceted functions, from cellular signaling to inducing cellular damage. Understanding these oxidants is crucial for translating research findings into therapeutic applications in redox medicine. ROS can be broadly categorized into radicals, such as one‐electron (radical) ROS, and non‐radicals, two‐electron ROS. Examples of one‐electron ROS include the superoxide anion radical (${O_{2\cdot }}^{-}$), which reacts with various molecules, the hydroxyl radical (HO·) causing significant biomolecular damage, peroxyl radical (ROO·) formed during peroxidation, and alkoxyl radical (RO·) with limited evidence for direct signaling. Non‐radical (two‐electron) ROS examples involve hydrogen peroxide (H_2_O_2_), singlet molecular oxygen (^1^O_2_), triplet carbonyls (RR'C=O*), and ozone (O_3_), each playing distinct roles in cellular processes, from signaling to causing damage in surface organs. Understanding these ROS categories is crucial for leveraging their therapeutic potential while mitigating their detrimental effects.^5^ Furthermore, it is important to note that ROS play a central role in interactions involving inflammation and metabolic control. Their excessive production can contribute to insulin resistance and impaired insulin secretion, creating a detrimental cycle. Disturbances in the redox system are characterized by significantly increased ROS production, leading to oxidative stress (OS), further exacerbating the cycle of metabolic dysfunction.^6^ This interplay emphasizes the critical nature of comprehending ROS mechanisms in addressing conditions related to metabolic disorders and inflammation.^6^

There is evidence to suggest that OS and inflammation play a role in the development of insulin resistance, which is a key feature of T2D.^4^, ^7^ They activate stress kinases like c‐Jun N‐terminal kinase (JNK) and the inhibitor of nuclear factor‐κB (IκB) kinase (IKK), hindering insulin receptor substrate‐1 (IRS‐1) and impairing insulin signaling. Additionally, these factors negatively affect adipose tissue, reducing adiponectin expression and secretion, further disrupting insulin signaling.^7^, ^8^

OS plays a pivotal role in the pathogenesis of various target organs affected by diabetes. In diabetic kidney disease (DKD), hyperglycemia triggers pathways such as renal hypoxia, altered tubulo‐glomerular feedback, and lipotoxicity, resulting in chronic inflammation and impaired kidney function.^9^, ^10^, ^11^, ^12^ When exposed to ROS, chronic inflammation, and OS, liver function can be impaired, resulting in fibrosis and cirrhosis. Inflammatory cytokines activate immune cells, generating ROS and causing oxidative damage.^13^ Similarly, chronic inflammation and OS are significant factors in heart disease, impacting cardiac function and contributing to the development of atherosclerosis and atrial fibrillation.^14^ Lastly, chronic inflammation in the brain is linked to neurological damage and mental health disorders, emphasizing the systemic impact of OS and inflammation in diabetes and the need for comprehensive management strategies.^15^

To ensure a comprehensive understanding of the relationship between inflammation and diabetes, we meticulously designed our literature search strategy to capture the most relevant and recent advancements in the field. We conducted our search in PubMed, utilizing a carefully selected set of keywords: “inflammation,” “diabetes,” “type 2 diabetes,” “type 1 diabetes,” “insulin resistance,” “ROS and diabetes,” “OS and diabetes,” “inflammation and diabetes,” and “inflammation and obesity.” Our search was specifically tailored to include articles published from January 2010 to December 2023, aiming to encompass over a decade of research advancements. This time frame was chosen to focus on the latest insights and developments in the understanding of the interplay between inflammation and diabetes, reflecting significant shifts in clinical approaches and therapeutic strategies observed during this period. The search was limited to articles published in English, encompassing both clinical and observational studies, to ensure the review's relevance and accessibility to the international scientific community. This strategy enabled us to synthesize the most current knowledge on the subject, while also delineating the evolution of research trends and treatment modalities over the past decade.

This review provides an overview of the current knowledge about diabetes and inflammation and examines various treatments that may prevent inflammation and, thus, prevent complications related to diabetes.

## 2 INFLAMMATION AND TYPE 2 DIABETES

**FIGURE 1:**
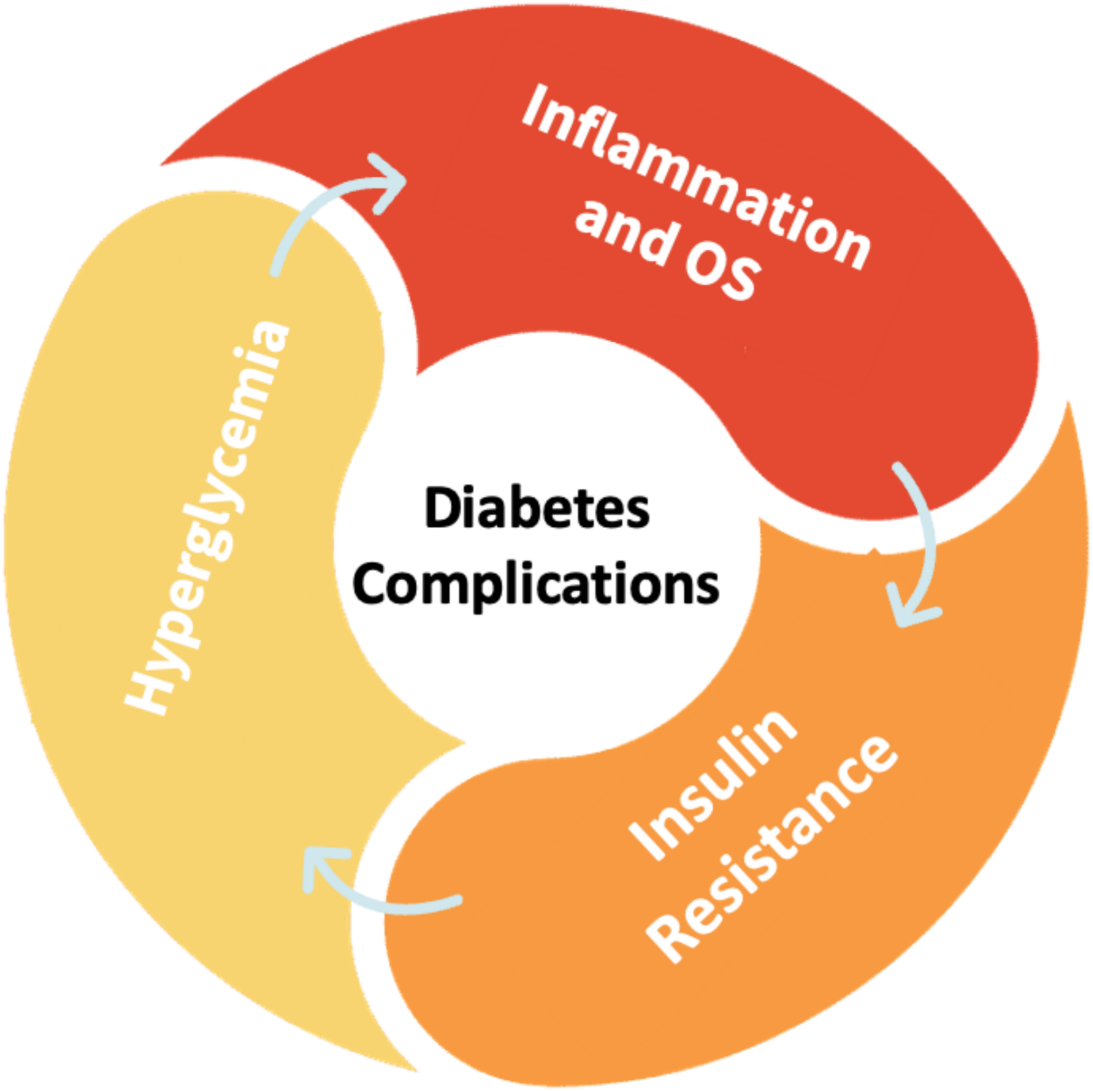
The vicious cycle of inflammation and hyperglycemia. Inflammation and OS contribute to insulin resistance, leading to high blood sugar levels (hyperglycemia). Simultaneously, elevated glucose levels trigger chronic inflammation. This vicious cycle between inflammation, OS, insulin resistance, and hyperglycemia escalates the risk of diabetes‐related complications.

Persistent inflammation and heightened OS are implicated in the development of insulin resistance, subsequently fostering hyperglycemia. Conversely, elevated glucose levels generate free radicals, thereby initiating chronic inflammation. This interplay between inflammation, OS, insulin resistance, and hyperglycemia perpetuates a detrimental cycle, elevating the risk of diabetes‐related complications^4^ (Figure 1).

Inflammation is an important physiological response of the body to various pathological processes. Hyperglycemia is one of the conditions that promote the release of inflammatory mediators. It leads to infiltration and subsequent activation of the cells of the innate and adaptive immune systems to the site of injury and to the production of inflammatory mediators.^3^ It promotes the creation of advanced glycation end‐products, activation of protein kinase C, and hyperactivity of hexosamine and sorbitol pathways. It also decreases the destruction or/and increases the production of enzymatic/non‐enzymatic catalase, superoxide dismutase, and glutathione peroxidase antioxidants, leading to the development of insulin resistance, impaired insulin secretion, and endothelial dysfunction by inducing excessive ROS production.^4^

- Nitric oxide (NO) plays a pivotal role as a molecule generated by endothelial cells. In addition to its function in vasodilation and antiplatelet effects, NO exhibits crucial properties. It possesses anti‐inflammatory effects within the endothelium, thereby aiding in immune response modulation and diminishing inflammation in blood vessels. NO also showcases antioxidant properties by neutralizing harmful free radicals; thereby alleviating OS within blood vessels. Vascular endothelial dysfunction refers to compromised endothelial function, resulting in reduced NO production and availability, among other issues. Various factors contribute to endothelial dysfunction, including OS, ROS, inflammation, insulin resistance, diabetes, hypertension, and aging.^16^, ^17^
- OS has been demonstrated to participate in the progression of diabetes. It plays a key role in diabetes, by hindering the function of insulin and increasing the incidence of disease complications. Recent evidence suggests it may also be important in contributing to vascular complications of diabetes through OS, inflammation, prothrombotic events, and endothelial dysfunction.^18^
- Cytokines can provoke OS by activating macrophages, which have a key function in removing the pathogens via the generation of ROS. In chronic inflammation related to T2D, macrophages change their phenotype from predominantly anti‐inflammatory M2‐type to increased proportions of pro‐inflammatory M1‐type. This is crucial to the initiation and amplification of islet inflammation.^19^
  It is important to note that chronic inflammation is a prolonged pathological condition characterized by tissue destruction and fibrosis, which culminates in cell damage due to overproduction of ROS from inflammatory cells.^20^ Endogenous antioxidant defense systems help to reduce the accumulation of deleterious ROS. Antioxidant enzymes can accelerate the breakdown of ROS, while the non‐enzyme antioxidants can capture and eliminate free radicals.^21^
  There is extensive evidence to suggest that inflammation plays a significant role in the development of insulin resistance. Studies have shown that chronic, low‐grade inflammation is a characteristic feature of obesity and metabolic disorders, including insulin resistance and type 2 diabetes. Inflammatory cytokines, such as tumor necrosis factor alpha (TNF‐α) and interleukin‐6 (IL‐6), have been shown to impair insulin signaling and glucose uptake in muscle and adipose tissue and to promote hepatic gluconeogenesis and lipolysis.^21^ In addition, the activation of toll‐like receptor 4, a key mediator of the innate immune response, has been implicated in the development of insulin resistance by promoting inflammation and OS.^22^ Several mechanisms have been proposed to explain the link between inflammation and insulin resistance. One hypothesis is that inflammatory cytokines can activate stress kinases such as JNK and inhibitor of IKK, which can dephosphorylate and inhibit IRS‐1, a key mediator of insulin signaling.^23^ Another hypothesis is that chronic inflammation can lead to the accumulation of ROS, which can promote insulin resistance by impairing insulin signaling and damaging cellular structures. There is also evidence to suggest that inflammation may be a key factor in the development of insulin resistance in NAFLD, a condition that is strongly associated with metabolic disorders such as insulin resistance and type 2 diabetes.^24^
  Overall, the evidence suggests that chronic inflammation plays a significant role in the development of insulin resistance and metabolic disorders. Reducing inflammation through lifestyle interventions such as diet and exercise may be an effective strategy for preventing and managing these conditions.

## 3 ORGAN DAMAGE

### 3.1 Nephropathy

DKD and hypertension are the leading causes of chronic kidney disease in all high‐income and middle‐income countries. The burden of DKD translates to 30%–50% of all CKD and affects more than 285 million people worldwide. Data from the National Health and Nutrition Examination Survey (NHANES) estimated that the prevalence of DKD in the United States was 14.35% in 2020, with a projection of 16.7% by 2030. Although other diabetic complications are declining, the prevalence of DKD has remained steady over the last 20 to 30 years.^25^

A predominant etiological factor responsible for the development of DKD is hyperglycemia. Hyperglycemia activates multiple pathways that contribute to the chronic inflammation leading to renal dysfunction. These pathophysiological pathways include renal hypoxia, altered tubulo‐glomerular feedback, hypertension, podocyte injury, lipotoxicity, mitochondrial dysfunction, impaired autophagy, and dysregulation of tubular exchangers. The damage caused by hyperglycemia is due to glucose uptake by renal cells that is not mediated by insulin. In the presence of established hyperglycemia, the renal cells are overwhelmed with glucose, leading to diversion of glucose to non‐glycolytic pathways. The result is the creation of advanced glycation end‐products (AGE), increased OS, and altered gene expression.^26^

Aldose reductase and hexosamine are two additional pathways related to hyperglycemia. Aldose reductase contributes to a proinflammatory environment which increases OS and further activates other pathways. The hexosamine pathway modifies proteins, resulting in elevated expression of transcription factors, including TGF‐β, Sp1, and plasminogen activator inhibitor‐1, creating a proinflammatory environment and increasing OS. AGE are a predominant factor in establishing chronic inflammation in the renal environment and with chronic exposure lead to DKD and renal failure. AGE cross‐link to structural proteins by engaging receptor for advanced glycation end‐products (RAGE). The linking initiates intracellular pathways that result in changes in gene expression and a striking upregulation of chronic inflammation. Hyperglycemia has a direct effect on the mitochondrial flux of glucose. The result is similar to the aldose reductase pathway, with an increase in OS leading to protein and DNA damage, further contributing to the inflammatory process.^9^, ^26^

### 3.2 Retinopathy

Diabetic retinopathy is a prevalent microvascular complication of diabetes. It is the leading cause of preventable vision loss in the elderly. Traditionally, it is classified as non‐proliferative diabetic retinopathy in its early stages and proliferative diabetic retinopathy or diabetic macular edema as the disease progresses. Hyperglycemia has a key role in causing microvascular damage and inflammation leading to retinal damage. An established state of hyperglycemia leads to OS and neurodegeneration via various pathways, resulting in retinal ischemia and detachment, vitreous hemorrhage, and neovascularization. The pathogenesis of micro‐vasculopathy and inflammation involves metabolic pathways like the creation and accumulation of AGE; the hexosamine pathway; the diacylglycerol pathway (protein kinase C pathway), resulting in altered gene expression; and the polyol pathway, resulting in OS. The chronic inflammatory state involves increased expression and upregulation of acute phase proteins, pro‐inflammatory cytokines, and chemokines. Notably, vascular adhesion molecules such as vascular cell adhesion molecule‐1; selectins such as E‐selectin; cytokines such as IL‐6, IL‐2β, and TNF‐α; chemokines such as monocyte chemotactic protein‐1 (MCP‐1); and integrins such as αvβ3, αvβ5, α5β1, and α5β3 have crucial roles in the recruitment, migration, and activation of inflammatory cells.^10^, ^27^, ^28^

### 3.3 Liver damage

Chronic inflammation and OS are closely linked and can impair liver function, leading to liver damage, fibrosis, and cirrhosis. Inflammatory cytokines, such as TNF‐α, IL‐6, and IL‐1β, can activate immune cells in the liver, leading to the production of ROS and OS. ROS can damage cellular structures, including proteins, lipids, and DNA, leading to cell death and inflammation. OS can also impair liver function by interfering with normal metabolic processes. Chronic liver disease, such as NAFLD, is associated with increased inflammation and OS. Viral infections, such as hepatitis B and C, can also lead to liver damage through inflammation and OS. Reducing inflammation and OS in the liver may be an important strategy for preventing and treating liver disease.^13^, ^29^

### 3.4 Heart damage

Chronic inflammation and OS have been identified as significant factors in the development and progression of heart disease. There is a growing body of research investigating the complex interplay between inflammation, OS, and cardiovascular disease. One study discussed the intricate connection between inflammation, OS, and the development of atherosclerosis and other cardiovascular diseases.^14^ The authors suggested that OS and inflammation have key roles in the pathogenesis of these conditions. Another study found that increased OS led to impaired cardiac function and increased the risk of heart failure in mice. The study suggested that OS plays a critical role in the development of atrial fibrillation, a common arrhythmia that can lead to heart failure. The authors proposed that antioxidants may be a promising therapy for this condition. These studies provide evidence for the close relationship between inflammatory OS and the heart and suggest that antioxidants may be a viable therapy for cardiovascular diseases.^14^, ^30^, ^31^, ^32^

### 3.5 Brain damage

Studies connect chronic inflammation to neurological damage. Two recent studies discussed the effect of chronic inflammation on depression and attention deficit hyperactivity disorder (ADHD).^15^, ^33^

Chronic inflammation caused by poor diet, unhealthy lifestyles, and exposure to toxins is becoming increasingly common and is a significant risk factor for chronic illness. Chronic inflammation can lead to depression by causing neuroinflammation and altering essential neural circuits, but the relation is complex. Prolonged, elevated inflammation can break down the barrier between the body and the brain, causing a leaky blood–brain barrier, allowing inflammation to pass into the brain and alter the neurons responsible for behavior and identity. A whole‐body approach is needed to manage depression, given the involvement of numerous biological systems alongside systemic inflammation.^15^

The systemic immune inflammation index and peripheral hemogram‐related inflammatory markers were used to investigate the potential role of neuroinflammation in adults with ADHD. The clinical symptoms and severity of ADHD were significantly correlated with inflammation. Hyperactivity scores were positively correlated with the systemic immune inflammation index and negatively correlated with platelets. Regression analysis revealed that the platelet index significantly predicted attention deficit. The study demonstrated the importance of inflammatory assessments specific to clinical presentations and the critical role of platelets in inflammatory processes in ADHD, which can be easily accessed from the results of a complete blood count.^33^

## 4 TREATMENT

**FIGURE 2:**
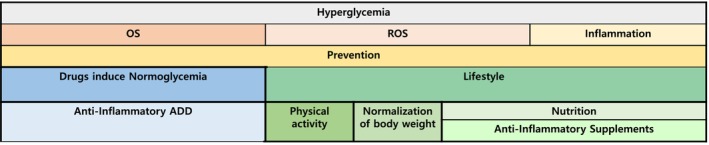
The relationship between OS and diabetes is well established. The use of healthy lifestyle, supplements, and ADD has a role in the reduction of OS and prevention of ROS and inflammation.

OS, ROS, and inflammatory processes play pivotal roles in the complications associated with diabetes, that cause damage to target organs. Inflammation can be mitigated by maintaining a healthy lifestyle, adopting a diet abundant in antioxidants (Mediterranean green diet), maintaining appropriate body weight, and engaging in regular physical exercise. Several dietary constituents, including flavonoids, carotenoids, curcumin, and gallic acid, among others, exhibit the potential to prevent OS and inhibit the development of ROS, thus averting diabetes‐related complications. Additionally, specific anti‐diabetic medications, such as metformin, TZD, and glucagon‐like peptide‐1 (GLP‐1) agonists, may exert a dual effect by regulating glucose levels and reducing inflammation (Figure 2).

### 4.1 Lifestyle

#### 4.1.1 Physical activity and normalization of body weight

There is abundant evidence in the literature regarding the beneficial effects of diet and exercise on the prevention and treatment of T2DM. Nevertheless, these effects are thought to be the sum of multiple mechanisms that are intricately connected, one of which is the effect on inflammation.^34^, ^35^

Dietary choices can either exacerbate or mitigate inflammation. The effect on inflammation is usually quantified by different inflammatory markers, including C‐reactive protein (CRP), TNF‐α, IL‐1β, IL‐4, IL‐6, and IL‐10 among others. Several studies have shown that a Mediterranean diet rich in fruits, vegetables, whole grains, olive oil, and lean protein sources can reduce inflammation and metabolic diseases. On the other hand, a diet high in saturated and trans fats, refined sugars, and processed foods can promote inflammation and metabolic diseases. Additionally, specific foods, such as omega‐3 fatty acids found in fatty fish and polyphenols found in colorful fruits and vegetables, have been shown to have anti‐inflammatory properties.^36^, ^37^

Nevertheless, it is not yet entirely understood whether the inflammatory response to diet choices is a direct cause of metabolic disease is or merely associated with it. Exercise can independently improve insulin sensitivity and glycemic control in patients with prediabetes or frank T2DM.^34^, ^38^, ^39^ In addition, cardiorespiratory fitness and muscular strength are inversely associated with the risk of developing T2DM, with cardiorespiratory fitness having a linear dose–response relationship.^40^

The anti‐inflammatory effects of exercise are thought to be beneficial for limiting metabolic disease. These effects are demonstrated by a decrease in the production of pro‐inflammatory cytokines, including CRP, TNF‐α, interleukin (IL) 6, and fibrinogen.^41^ Even a single exercise session can increase anti‐inflammatory markers such as IL‐10, IL‐1 receptor antagonist, and muscle‐derived IL‐6.^42^ Moreover, exercise can improve the body's endogenous antioxidant capacity by increasing the enzymatic activity of glutathione peroxidase and glutathione reductase, as well as downregulating OS mediating gene expression.^43^

In addition to proper nutrition and exercise, maintaining a healthy body weight and avoiding smoking and excessive alcohol consumption can also help prevent inflammation. Visceral fat is recognized as a significant contributor to inflammation. Consequently, abdominal obesity is considered a primary cause of inflammation in target organs, often associated with the presence of metabolic syndrome in individuals with diabetes. Fat accumulation in organs such as the liver and heart can exacerbate inflammation.^44^ Adopting a healthy lifestyle, which includes adhering to a Mediterranean or a green diet, represents one approach to preventing excessive fat deposition. These dietary patterns have been scientifically proven to assist in maintaining a healthy body weight.^44^, ^45^ Overall, a healthy lifestyle that includes a balanced diet and regular exercise is crucial for preventing chronic inflammation and for reducing the risk of associated diseases.^37^, ^41^, ^46^, ^47^, ^48^

Exogenous antioxidants can be acquired through diet and through supplements. Various antioxidants and other dietary components may have additive or synergetic effects that may not manifest in studies that look at interventions with only one substance, such as a specific antioxidant. This assumption was examined in the Rotterdam study, a population‐based cohort with 15 years of follow‐up. It concluded that higher total dietary antioxidant capacity (as measured by ferric reducing ability of plasma) in adults is associated with a lower incidence of T2D (HR = 0.84, CI = 0.75–0.95; *p* = 0.01), but not prediabetes (HR = 0.93, *p* = 0.13, CI = 0.84–1.02), and showed a decrease in insulin resistance (as measured by the homeostasis model assessment of insulin resistance). Most of the dietary antioxidants in the study came from common foods such as coffee, fruit, vegetables, tea, and chocolate.^49^

Another study found an inverse linear association between total antioxidant capacity and the risk of T2D among women^50^ and further strengthens the assumption that dietary antioxidants may be beneficial in relation to T2D. The various ingredients that may affect inflammation will be discussed.

#### 4.1.2 Nutrition—Anti‐inflammatory supplements

##### Flavonoids

Flavonoids are a class of polyphenolic compounds found in many plant‐based foods, including fruits, vegetables, and tea. These compounds have been studied extensively for their potential health benefits, including their ability to reduce inflammation and OS.^51^

Flavonoids exhibit anti‐inflammatory effects through several distinct mechanisms, each contributing to a decrease in inflammation and to potential health benefits. First, they inhibit regulatory enzymes, such as protein kinases and phosphodiesterases, leading to decreased signal transduction and diminished cell activation. Additionally, flavonoids possess antioxidant properties. They act as scavengers and inhibit the production of free radicals, resulting in a decrease in OS. Furthermore, these compounds impact arachidonic acid metabolism by inhibiting enzymes like PLA2, COX, and LOX, which in turn reduces the release of proinflammatory mediators such as prostaglandins, thromboxanes, and leukotrienes. Flavonoids also modulate gene expression by targeting transcription factors like NF‐κB, GATA‐3, and STAT‐6, resulting in decreased transcription of proinflammatory genes. Lastly, these compounds have a profound impact on immune cells, inhibiting their activation, maturation, signaling transduction, and secretory processes.^52^

In addition to their potential role in reducing inflammation and OS, flavonoids have been associated with numerous other health benefits in in vitro and animal studies, including improved cardiovascular health, cognitive function, food allergy, asthma, inflammatory bowel disease, and a reduced risk of certain types of cancer.^52^, ^53^, ^54^

Specifically, flavonoids may also improve glucose metabolism through various mechanisms. In vitro studies have demonstrated their ability to increase glucose uptake through AMPK phosphorylation and promotion of GLUT4 translocation, to decrease gluconeogenesis through inhibition of PEPCK and G6P, and to enhance insulin signaling transduction. In vivo experiments further support these findings; illustrating that flavonoids contribute to the improvement of insulin sensitivity in skeletal muscle, adipose, and hepatic tissue. These effects were also demonstrated in clinical trials where flavanols improved insulin sensitivity and decreased HbA1c.^55^

In a study that included patients with metabolic syndrome, the flavonoid, quercetin, reduced inflammation, OS, and systolic blood pressure.^51^ Most research conducted on flavanols consists of in vitro and animal studies. Despite yielding promising results, there is a notable scarcity of clinical human trials. As a result, while a diet high in flavonoids promotes health, developing specific therapies with specific flavonoids and accurate dosages is not yet possible due to the need for more human data in general, and randomized controlled trials (RCTs), specifically.

##### Carotenoids

Carotenoids are a class of naturally occurring lipophilic pigments that create the characteristic colors of some fruits and vegetables and are widely distributed in the plant kingdom and certain microorganisms.^56^ The primary biological function of carotenoids in plants is to participate in photosynthesis, where they serve as light‐absorbing pigments, transferring absorbed energy to chlorophyll molecules for the conversion of light into chemical energy.^57^ Nevertheless, in the context of human physiology, carotenoids serve as both precursors to vitamin A and antioxidants.^58^

The main carotenoids identified in the plasma of individuals who ingest foods abundant in carotenoids include lycopene, α and ß‐carotene, lutein, zeaxanthin, and ß‐cryptoxanthin.^59^ Carotenoids possess both antioxidative and anti‐inflammatory properties. The antioxidative effects are both direct, by scavenging of free radicals, and indirect, by interacting with cellular signaling pathways including nuclear factor κB (NF‐κB), mitogen‐activated protein kinase (MAPK), or nuclear factor erythroid 2–related factor 2 (Nrf2).^60^ In a meta that examined the effects of carotenoids supplementation on OS, supplementation was associated with significantly increased levels of antioxidative parameters including ferric‐reducing ability of plasma (FRAP) and oxygen radical absorbance capacity (ORAC).^61^ In a different meta‐analysis of randomized controlled trials (RCTs), carotenoid supplementation reduced C reactive protein (CRP) (WMD: −0.54 mg/L, 95% CI: −0.71 to −0.37) and interleukin‐6 (IL‐6) (WMD: −0.54 pg./mL, 95% CI: −1.01 to −0.06), but not tumor necrosis factor α (TNF‐α).^62^

The ability of carotenoids to mitigate OS and the damage it causes has sparked interest in its use for various diseases and specifically in T2DM. In observational studies, elevated serum levels of carotenoids have demonstrated an inverse association with the incidence of T2DM.^63^, ^64^

This observation was subsequently reinforced through a meta‐analysis, revealing a negative correlation between total carotenoid levels and the metabolic syndrome; this association was strongest for β‐carotene specifically.^65^ However, in a long‐term randomized controlled trial involving over 20 000 participants, supplementation with β‐Carotene did not result in a reduction in the incidence of T2DM.^66^ Moreover, diminished serum carotenoid levels constitute a risk factor for obesity, and supplementation has been linked to a reduction in body weight, thereby reinforcing its potential efficacy in individuals with T2DM.^67^ Increasing dietary carotenoids in general, and β‐carotene specifically, might be useful in the prevention and treatment of T2D to some degree, perhaps due to its antioxidant effects.^68^

Further research, including RCT with hard outcomes related to T2D, is needed to substantiate these assumptions and to make further recommendations.^68^, ^69^

##### Curcumin

Turmeric has been used in Indian culture for centuries, and today, it is used worldwide. Curcuminoids, a class of polyphenols naturally found in turmeric, are primarily responsible for the pharmacological activity associated with turmeric and specifically the curcuminoids curcumin, desmethoxycurcumin, and bisdemethoxycurcumin.^70^ Curcumin is associated with a multitude of antioxidant, anti‐inflammatory, anti‐angiogenic, and anticancer properties.^71^

In clinical trials, supplementation with curcumin consistently reduces malondialdehyde (MDA), a marker of OS, compared to placebo. Additionally, an association between supplementation and elevated levels of antioxidative enzymes, such as superoxide dismutase (SOD), catalase (CAT), and glutathione peroxidase (GPx), has been noted, although the findings in this regard are inconsistent.^71^, ^72^, ^73^ In the context of inflammation, various meta‐analysis of RCTs investigated the effects curcumin supplementation and found a statistically significant reduction in different inflammatory markers including CRP, iL‐1, IL‐6, and TNF‐α, although yet again some inconsistency was noted.^73^, ^74^, ^75^ Owing to the role of inflammation and OS in the pathophysiology of T2DM, the antioxidative and anti‐inflammatory properties of curcumin may potentially impede the development and progression of T2DM and its sequalae. In a RCT of 240 prediabetic patients, curcumin supplementation resulted in fewer patients progressing to frank diabetes after 9 months of treatment compared to placebo (0% vs. 16.4%, respectively). The curcumin group also demonstrated lower insulin resistance and CRP levels.^76^

In another RCT, curcumin supplementation improved and reduced the severity of peripheral neuropathy in T2DM patients, as well as reduced HbA1c.^77^ In a meta‐analysis of RCTs that included T2DM patients, supplementation reduced HbA1c (weighted mean difference [WMD]: −0.69%; 95% CI: −0.91 to −0.48), as well as improved insulin resistance and lipid profile in some populations.^78^

The safety of curcumin supplementation is well established, even in high doses, and it is considered safe by the US Food and Drug Administration.^74^ Current data from clinical trials and non‐clinical studies suggest that curcumin supplementation may be a beneficial addition to current diabetes treatment and its prevention. Nevertheless, RCTs that examine the addition of curcumin to current treatment and its combined effects are needed before further recommendations can be made.

##### Gallic acid

Gallic acid (GA) is a naturally occurring phenolic compound found in many fruits, vegetables, and medicinal plants. In animal and in vitro studies, this compound displayed a diverse spectrum of biological activities, including antioxidative, antimicrobial, anti‐inflammatory, anticancer, and beneficial metabolic properties.^79^, ^80^

GA features free radical scavenging and antioxidant abilities through different mechanisms. Mainly due to its phenolic hydroxyl group, GA can interact with different ROS molecules directly and limit their production of free radicals, thus mediating oxidative damage. Additionally, GA plays a role in the synthesis of different enzymes in cellular redox pathways and may increase their levels and activity, including SOD, CAT, reduced glutathione, and GPx.^79^, ^81^ Potentially, the combination of these effects may reduce lipid and protein peroxidation, as well as DNA damage, consequently inhibiting the inflammatory response and tissue injury.

In the realm of metabolic disorders, GA exhibits inhibitory effects on diet‐induced hyperglycemia and hypertriglyceridemia, reduces adipocyte size, and safeguards pancreatic β‐cells. Mechanistically, these actions are mediated through the induction of nuclear transcription factor PPAR‐γ that promotes differentiation and insulin sensitivity in adipocytes. GA further enhances cellular glucose uptake by facilitating the translocation of insulin‐stimulated glucose transporters such as GLUT1, GLUT2, and GLUT4 through the activation of the phosphatidylinositol 3‐kinase (PI3K)/p‐Akt signaling cascade. GA may enhance the activity of hepatic glycolytic enzymes, including hexokinase, aldolase, and phosphofructokinase, while also inhibiting the activity of hepatic gluconeogenic enzyme fructose‐1,6‐bisphosphatase.^82^

In a placebo‐controlled clinical trial involving 19 participants with type 2 diabetes, GA intake significantly reduced oxidative DNA damage and lowered inflammation markers, including oxidized‐LDL and C‐reactive protein.^83^ Overall, GA shows promise due to its significant antioxidant activity and ability to reduce OS and related damage to cells and tissues. However, most data on GA is based on animal and in vitro studies and outcome‐oriented clinical human trials are scarce. Further studies are needed to investigate its full potential as a therapeutic agent for OS‐related disorders.

##### Green tea

Green tea (GT), derived from the leaves of *Camellia sinensis*, is one of the world's oldest and most widely consumed beverages. GT contains various biologically active compounds, with catechins, a group of flavonoids, as the primary antioxidant agents. The catechins in GT include epicatechin‐3‐gallate, epigallocatechin, epicatechin, and the well‐known epigallocatechin‐3‐gallate.^84^ The catechin group is primarily recognized for its significant antioxidant, anti‐inflammatory, and cancer prevention properties, highlighting its biological activities.^84^

The antioxidative effects of catechins in GT include the induction of antioxidative enzymes such as SOD, CAT, and GPx, scavenging of ROS, as well as inhibiting the formation of free radicals and lipid peroxidation.^85^ In a recent systematic review of RCTs by Rasaei et al., GT supplementation was found to improve total antioxidant capacity and may reduce MDA levels, a product of lipid peroxidation, in a dose–response manner.^86^ However, a systematic review and meta‐analysis by Asbaghi et al. found that among patients with T2D specifically, GT supplementation did not affect total antioxidant capacity or MDA levels. Nevertheless, it was noteworthy that the supplementation led to a reduction in CRP levels, suggesting a potential influence on systemic inflammation in this particular population (WMD: −5.51 mg/dL, 95% CI: −9.18 to −1.83, *p* = 0.003).^87^ GT supplementation may also have anti‐obesogenic properties. A systematic review and dose–response meta‐analysis of RCTs that investigated the effects of GT on obesity found that GT supplementation was associated with a reduction in body weight (WMD: −1.78 kg, 95% CI: −2.80 to −0.75, *p* = 0.001) and body mass index (WMD: −0.65 kg/m^2^, 95% CI: −1.04 to −0.25, *p* = 0.001).^88^

These effects were also demonstrated in T2DM patients, as well. Yet, this effect may be in part due to the caffeine content in GT, which is known to promote weight loss.^89^, ^90^ In a systematic review and meta‐analysis of RCTs investigating the impact of GT supplementation on glycemic control, the pooled results demonstrated a statistically significant reduction in fasting blood glucose levels, with a mean decrease of −1.44 mg/dL (95% CI: −2.26 to −0.62 mg/dL; *p* < 0.001) with minimal heterogeneity (*I* ^2^ = 7.7%) in this outcome. However, GT consumption did not yield statistically significant changes in fasting insulin and HbA1c values.^91^ A meta‐analysis that examined the effect of GT on insulin resistance and glycemic control in T2DM patients strengthens these findings, as no difference was found between GT and placebo groups in HbA1c, homeostatic model assessment for insulin resistance (HOMA‐IR) fasting insulin, or fasting glucose.^91^

In summary, GT supplementation is associated with numerous beneficial effects; yet, the clinical significance of this intervention is not clear. Nevertheless, GT supplementation may prove in the future to be a valuable tool in addition to the T2DM prevention and treatment therapies currently in use.

##### Vitamins and minerals

Vitamins are organic compounds that cannot be synthesized by an organism but are essential in small quantities for proper function and development. Vitamins exhibit antioxidant properties by various mechanisms, including enhancing the efficacy of antioxidative enzymes, serving as co‐enzymes in their physiologically reduced states, or by directly engaging with free radicals. Vitamins C, E, and carotenoids are widely recognized for their antioxidant properties; however, other vitamins also demonstrate varying degrees of antioxidative properties.^92^, ^93^

There is conflicting evidence regarding the use of vitamin supplements in the context of T2DM. There is considerable heterogenicity between studies, as well as inconsistent results, leading to uncertainty about the utility of this intervention. A recent systemic review and meta‐analysis that investigated the effects of vitamin C supplementation on glycemic control in patients with T2DM found a significant decrease in HbA1c (WMD: 0.51%, 95% CI: 0.81 to 0.20; *p* = 0.001), fasting insulin, and fasting blood glucose levels with vitamin C supplements compared to untreated patients. Furthermore, a linear dose–response relation was found with changes in HbA1c. Yet, the studies were very heterogeneous (*I* ^2^ = 80.9%–90.4%).^94^ Another systemic review and meta‐analysis of RCTs found that vitamin C supplementation was associated with reduced HbA1c (−0.54% CI: −0.90 to −0.17; *p* = 0.004), and systolic and diastolic blood pressure, but again with high heterogeneity between studies (*I* ^2^ > 50%) and very low certainty of evidence.^95^

Vitamin E does not seem to help glycemic control in most T2DM patients but may improve HbA1c in patients with inadequate glycemic control (HbA1c >8%) or low serum levels of vitamin E.^96^ Vitamin E may also improve prognosis of diabetic nephropathy in subsets of patients.^97^ Combination therapy with vitamin E may improve non‐alcoholic steatohepatitis in T2DM patients.^98^ Although one meta‐analysis suggested that high doses of vitamin E may increase mortality, this finding has been criticized.^99^, ^100^

The overall use of vitamins and antioxidants may improve conduction velocity of nerves in T2DM patients with diabetic peripheral neuropathy.^101^ In a systemic review and meta‐analysis of RCTs that investigated antioxidant effects of vitamins in T2DM, vitamin E demonstrated a notable decrease in both fasting blood glucose levels and glycated HbA1c in comparison to a placebo. Concurrently, vitamins C and E were associated with the reduction of MDA and thiobarbituric acid reactive substances, which are the product of lipid peroxidation and are used to evaluate OS. In addition, augmentation with GPx, SOD, and total antioxidant capacity relative to a placebo was observed. Nevertheless, inconsistencies persist in the outcomes reported, potentially attributable to a lack of standardized measures.^102^

Minerals are inorganic compounds and essential micronutrients for a variety of metabolic processes. Some minerals, such as zinc, copper, selenium, and magnesium, exhibit antioxidant properties.^103^ Zinc is a co‐factor for a myriad of enzymes, including some of those with antioxidant activity, such as SOD. It is involved in glutathione synthesis, which directly neutralizes free radicals.^104^ People with diabetes frequently have lower amounts of zinc compared to the non‐diabetic population.^105^ A systemic review and meta‐analysis of RCTs found that zinc supplementation improved glycemic control compared to placebo, as measured in a fasting blood glucose, fasting insulin, homeostasis model assessment of insulin resistance (HOMA‐IR), HbA1c (WMD: −0.55%; 95% CI: −0.84 to −0.27%), and even improved CRP levels.^106^

Magnesium is involved in various intracellular processes across multiple tissues and plays a role in glucose metabolism. Existing evidence suggests that insulin serves as a crucial regulatory factor for the intracellular accumulation of magnesium. Upon entry into the cell, magnesium functions as a second messenger in facilitating insulin action, particularly in the context of oxidative glucose metabolism.^107^ Notably, hypomagnesemia has been identified in individuals with T2DM, particularly those with inadequate glycemic control.^108^ Nevertheless, in a systemic review and meta‐analysis of RCTs, oral magnesium supplementation failed to improve glycemic control in T2DM patients.^109^

In conclusion, although current data indicate that vitamins and minerals could play a role in the prevention and management of T2DM, due to conflicting evidence and the quality and lack of standardization of current data, we cannot at this time form a strong conclusion regarding vitamin and mineral supplementation as a form of therapy for T2DM.

### 4.2 Drugs induce normoglycemia

#### 4.2.1 Anti‐inflammatory ADD

Ongoing research is evaluating whether anti‐diabetic drugs are possible anti‐inflammatory agents and the effects of anti‐diabetic drugs on various indicators of inflammation. The treatment is often based on a change in lifestyle along with the use of the drug, rather than a placebo.

##### Pioglitazone

Pioglitazone is a drug that activates the protein peroxisome proliferator‐activated receptor (PPAR)‐γ, which helps reduce insulin resistance in the body. In a study involving 34 patients with T2D, pioglitazone treatment for 6 months significantly reduced levels of circulating CRP but not IL‐6. Another meta‐analysis found that pioglitazone significantly lowered hsCRP levels in patients with type 2 diabetes but did not affect IL‐6.^110^, ^111^

##### SGLT‐2 inhibitors (SGLT‐2i)

In vitro studies and preclinical models have consistently demonstrated the capacity of SGLT‐2i to mitigate cytokine production and counteract multiple pro‐inflammatory pathways.^112^, ^113^ Intriguingly, clinical trials have corroborated these findings by documenting a decrease in levels of pro‐inflammatory cytokines and a concurrent downregulation of inflammatory immune cells subsequent to gliflozin treatment. Notably, recent investigations have postulated potential anti‐inflammatory effects stemming from elevated ketone bodies during SGLT‐2i therapy.^114^, ^115^ The collective evidence strongly suggests that both direct and indirect anti‐inflammatory mechanisms are intricately linked to the observed therapeutic benefits attributed to SGLT‐2i.^114^

##### Insulin

The recognition of insulin's anti‐inflammatory properties dates back to its ability to induce vasodilation through endothelial nitric oxide (NO) release in arteries, veins, and capillaries, thereby reducing leukocyte adhesion and infiltration. Additionally, insulin demonstrates inhibitory effects on platelet adhesion and aggregation. Studies have corroborated these observations by revealing that insulin suppresses crucial inflammatory mediators, namely intercellular cell adhesion molecular‐1 (ICAM‐1), MCP‐1 expression, and NFκB binding in human aortic endothelial cells in vitro. These suppressive effects, mediated by NO release, can be negated by the NOS inhibitor N(G)‐nitro‐L‐arginine. Among pro‐inflammatory cytokines, TNF‐α plays a pivotal role in inducing the production of other cytokines and expression molecules. Investigations in myocardial ischemia/reperfusion (I/R) rats demonstrated that insulin inhibits TNF‐α induction locally and systemically.^116^, ^117^ It was also revealed for the first time that insulin treatment in vitro attenuates I/R‐induced TNF‐α production in cardiomyocytes. Considering the importance of polymorphonuclear neutrophils (PMN) in defense against infection, experiments in a rabbit model revealed that insulin reduces P‐selectin and ICAM‐1 expression in endothelial cells, leading to decreased PMN adherence and I/R‐induced inflammatory injury. Furthermore, insulin's ability to alleviate the endotoxin‐induced systemic inflammatory response, by decreasing IL‐6 and TNF‐α expression while enhancing the anti‐inflammatory cascade in normoglycemic rat and porcine models, underscores its role as a potent anti‐inflammatory agent. These collective findings strongly support insulin's potential in alleviating inflammation by suppressing pro‐inflammatory cytokines and immune mediators.^116^

##### Metformin

Both basic and clinical evidence support an anti‐inflammatory effect of metformin. Metformin has been shown to reduce the secretion of proinflammatory cytokines in liver cells and macrophages. Several studies have reported that metformin is associated with lower CRP levels.^98^ However, some studies did not find that metformin affected CRP levels in patients with T2D. Meta‐analyses have shown that metformin can reduce CRP levels in middle‐age and older adults with chronic low‐grade inflammation, as well as in women with polycystic ovary syndrome.^110^, ^118^, ^119^, ^120^, ^121^

##### GLP‐1 agonist

The available data on the effects of GLP‐1 agonist on CRP and IL‐6 are limited. One study showed that 1.5 mg a week of dulaglutide significantly decreased hsCRP levels compared with placebo in 755 patients with T2D. Another study demonstrated a significant reduction in serum IL‐6 levels after 26 weeks of 0.75 or 1.5 mg dulaglutide, once a week.^122^

It also known that GLP‐1RA is associated with preventing damage to target organs, some of which are caused by inflammation. Cardiovascular outcome trials have demonstrated that treatment with GLP‐1RA is linked to substantial cardiovascular benefits. Real‐world studies investigating the cardio‐renal outcomes of GLP‐1RA have suggested that initiating GLP‐1RA treatment is associated with even greater benefits in terms of composite cardiovascular outcomes, major adverse cardiovascular events, all‐cause mortality, myocardial infarction, stroke, cardiovascular death, and peripheral artery disease.^123^

## 5 LIMITATIONS

The development of metabolic diseases, diabetes, and subsequent organ damage is associated with increase in OS, ROS, and inflammation. However, the effect of antioxidants and some ADD remains uncertain. While encouraging results have emerged from preliminary studies, translating these findings into practical clinical applications requires validation via RCTs. Furthermore, assessing the long‐term impact of antioxidants on disease progression, complications, and overall mortality in individuals with diabetes demands careful consideration of their efficacy.

## 6 CONCLUSION

In the modern world, chronic inflammation caused by poor nutrition, an unhealthy lifestyle, and exposure to toxins is becoming increasingly common. This poses a significant risk factor for chronic diseases. Evidence suggests that OS, ROS, and inflammation may play a role in the development of insulin resistance and β‐cell dysfunction. Studies on inflammation and type 2 diabetes indicate a vicious cycle: Chronic inflammation can contribute to insulin resistance, while hyperglycemia leads to OS and further promotes inflammation.

OS, ROS, and inflammation that increase deleterious biomarkers seem to play a significant role in the complications of diabetes and damage to the target organs. Preventing inflammation can be achieved by maintaining a healthy lifestyle, consuming a diet rich in antioxidants, maintaining proper body weight, and engaging in regular physical activity. Various dietary components, such as flavonoids, carotenoids, curcumin, and gallic acid, among others, as well as certain vitamins and minerals supplementation, may prevent OS and the development of ROS, thereby preventing inflammation and complications related to diabetes. Certain anti‐diabetic medications, such as insulin, TZD, SGLT‐2i, metformin and GLP‐1 agonists, have an effect on glucose regulation and reduction of inflammation.

Treatment that combines the noted lifestyle and dietary habits is usually more affordable than existing alternatives. It also has fewer side effects and, in our opinion, may have better compliance among certain populations that are averse to medication. While there is a significant amount of associated data on the prevention and treatment of diabetes with antioxidants, RCTs evaluating these interventions are lacking.

It is crucial to conduct these types of studies to evaluate the most promising interventions discussed in this review, in order to reach a consensus regarding their use.

## AUTHOR CONTRIBUTIONS

Manuscript writing: all authors; manuscript revision: Roni Weinberg Sibony and Itamar Raz; final approval of manuscript: all authors.

## FUNDING INFORMATION

This research received no external funding.

## CONFLICT OF INTEREST STATEMENT

The authors declare no conflicts of interest.
